# Vein Distribution on the Deformation Behavior and Fracture Mechanisms of Typical Plant Leaves by Quasi In Situ Tensile Test under a Digital Microscope

**DOI:** 10.1155/2020/8792143

**Published:** 2020-06-30

**Authors:** Jingjing Liu, Wei Ye, Zhihui Zhang, Zhenglei Yu, Hongyan Ding, Chao Zhang, Sen Liu

**Affiliations:** ^1^Faculty of Mechanical & Material Engineering, Huaiyin Institute of Technology, Huai'an 223003, China; ^2^The Key Laboratory of Engineering Bionic (Ministry of Education, China) and the College of Biological and Agricultural Engineering, Jilin University, 5988 Renmin Street, Changchun 130025, China

## Abstract

Angiosperm leaf venation is based on two major patterns, typically dicotyledonous branching and monocotyledonous parallel veins. The influence of these patterns on deformation and fracture properties is poorly understood. In this paper, three species of dicotyledons with netted venation and three species of monocots with parallel venation were selected, and the effect of vein distribution of leaves on their mechanical properties and deformation behavior was investigated. Whole images of leaves were captured using a digital camera, and their vein traits were measured using the image processing software *Digimizer*. A self-developed mechanical testing apparatus with high precision and low load was used to measure the tensile properties of leaves. The deformation behavior of the leaf was captured using a digital microscope during the tensile test. Results showed that the vein architecture of monocots and dicots is different, which had a remarkable effect on their mechanical properties, deformation behavior, and crack propagation behavior. The greater the diameter and the more the number of veins parallel to the tensile direction, the higher the tensile force, tensile strength, and elastic modulus of the leaves. The netted venation leaves evinced the elastic-plastic fracture type, and the hierarchy venation provided resistance to fracture propagation of cracks in the leaves by lengthening the crack path. The leaves with parallel venation behaved in a predominantly brittle manner or elastic fracture type, and the parallel venation inhibited the initiation of cracks in the leaves by increasing the load at complete fracture of the leaves. The investigation provides reference for a stiffened plate/shell structure and bionic anticrack design.

## 1. Introduction

In nature, plants have different leaf and vein structures which may be related to competing selection pressures on the leaf form and function influenced by potential phylogenetic constraints. The form and function of the leaf venation structure are important for plant photosynthesis and ultimately overall plant performance. Interpreting leaf shape has important implications for paleoecology, paleoclimatology, agriculture, urban ecology, and biomimetic optimization design [1–6]. In particular, plant venation presents various patterns and has attracted the attention of many botanists and physicists.

According to the distribution patterns in the leaves, veins can be divided into three types, dichotomous venation, parallel venation, and netted venation. *Ginkgo biloba* has distinctive fan-shaped leaves possessing an open dichotomous venation pattern that is common among ferns, but less research has been done on dichotomous venation [7]. Monocotyledonous leaves possess parallel or curved veins, and their main vein and lateral veins of the parallel venation are arranged in parallel in grass and *Canna indica L*. The netted venation of dicotyledonous plants is the most complex. In netted venation, the primary vein (1° vein) extends from the base to the tip of the leaf lamina. The 2° veins are defined as those that branched from the 1° veins, distinct in size and pattern from the 3° veins, which form the largest-gauge reticulate mesh in the leaf [2]. Global scaling relationships of venation traits with leaf size across dicotyledonous species indicated that larger leaves had major veins of larger diameter but lower length per leaf area, whereas minor vein traits were independent of leaf size [8, 9].

Leaf venation plays an important role in transport and mechanical support. Leaves, which are plate structures stiffened by venation, are valuable natural examples of stiffened plate structures and are worth investigating. In order to better understand the venation characteristics of leaves, the venation morphogenesis mechanism has been widely investigated. There are three basic areas to investigate morphogenetic mechanisms: auxin canalization [10, 11], mechanical force [12], and fluid transport [13, 14]. The distribution of leaf venation follows the physiological principle of minimum investment of structure to achieve the requisite function while ensuring that the vector sum of all forces is in equilibrium for a self-supporting leaf [12, 13], then a better understanding of venation helps us to apply biomimetics to solve similar engineering problems. For example, inspired by growth mechanism of leaf venation, Liu et al. [15] proposed an adaptive morphogenesis algorithm to design stiffened plate/shell structures in a growth manner, and this algorithm produced effective stiffeners along arbitrary directions.

Leaves of many species are thin, flat structures, susceptible to herbivores, wind, and other sources of physical damage [16]. Studies on how leaves achieve their structural integrity under complex natural conditions and the effect of different vein distribution on mechanical properties of leaves have attracted increasing attention. The mechanical properties of leaves have been revealed using punch, tear, bend, or shear types of techniques showing that highly scleromorphic leaves are an order of magnitude tougher and stronger than soft leaves [17–19]. Wang et al. [20] investigated the structures and mechanical properties of eight species of plant leaves using tensile and nanomechanical tests. The results indicated that the ultimate strength of a leaf is related to both the material composition and the structure, and the coriaceous leaves usually exhibit higher tensile strength. In a recent study on stiffness of leaf epidermis and mesophyll of 36 angiosperm species, Onoda et al. [21] demonstrated that the stiffness of epidermis layers is higher in evergreen species than in deciduous species and strongly associated with cuticle thickness.

Leaf venation geometry and density also influence the mechanical stability and mechanical behavior of a leaf [22]. Greenberg et al. [23] studied the tensile behavior of ryegrass indicating that the tensile properties depend on the location where the tensile test was run on the specimen and the strain rate of the tensile test. Whereas some of the grass leaf specimens behaved in a predominantly brittle manner, others evinced a semiductile mode. Lucas et al. [24] investigated the fracture toughness of the *Calophyllum inophyllum L.* leaf using cutting and notched tensile tests. The results show that toughness was found to depend on the presence of veins in the fracture path. Both tensile and cutting tests imply that the fracture path at right angles to secondary veins was 3.0 times more than that parallel to them. To survive, the plant must have mechanisms for resisting fracture (the initiation and propagation of cracks). Balsamo et al. [25] found that the tensile fracture planes differed between two species with *P. serrulata* fracturing along the secondary veins, while *H. arbutifolia* mainly fractured across the leaf irrespective of the vein or mesophyll position. When leaves of *P. serrulata* and *H. arbutifoli*a were subjected to tensile forces, the stress-strain curve produced was consistently of the elastic-plastic type. In tensile tests of leaves, Meidani et al. [26] also observed that when a crack tip propagated through a leaf and intersected a strong vein, the crack was deflected along the interface. However, few studies have focused on the effect of vein distribution on leaf mechanical properties, deformation, and crack propagation behavior.

In this paper, the venation morphology and tensile properties of leaves from six species of plants were studied, and the effects of vein distribution on leaf mechanical properties, deformation behavior, and crack propagation behavior were also investigated. First, three species of dicotyledons with netted venation and three species of monocots with parallel venation were selected in this study. A digital camera was used to capture whole images of leaves, and their vein traits were extracted using the image processing software *Digimizer*. Secondly, according to the distribution and orientation of veins, several typical test locations were taken from each leaf for quasi in situ tensile tests, and the deformation behavior of the leaf was captured using a digital microscope during the tensile test. Thirdly, combining the leaf traits and the crack propagation behavior through the leaf surface of different leaves, the effect of vein distribution on the deformation behavior and fracture mechanisms was analyzed and compared. This will give insights into designing a stiffened plate/shell structure and bionic anticrack materials.

## 2. Materials and Methods

### 2.1. Materials

**Figure 1:**
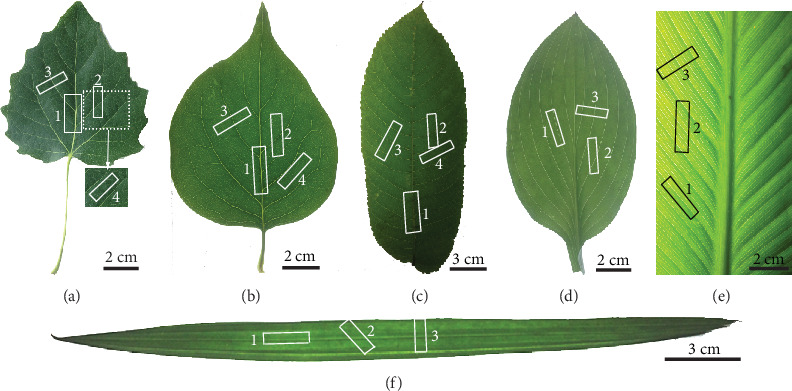
The morphology, location, and orientation of test pieces of leaves of three dicot and three monocot plant species used for tensile tests: (a) *P. alba*, (b) *S. oblata*, (c) *A. altissima*, (d) *H. plantaginea*, (e) *C. indica*, and (f) *R. excelsa*.

There is considerable variation in leaf venation around two major patterns within the angiosperms, including the branching and reticulate dicot pattern and the parallel monocot pattern. For reticular venation, it includes pinnate and palmate. In parallel venation of leaves, the veins are either strictly parallel, curved and approximately parallel, or penni-parallel (pinnate-parallel). A penni-parallel leaf has a central midvein with secondary veins that are essentially parallel to one another [27]. For parallel veins (parallelodromous), the parallel 1° veins originate collaterally at the leaf base and converge toward the leaf apex. For curved and approximately parallel veins (campylodromous), the parallel 1° veins originate collaterally at or near the leaf base and run in strongly recurved arches that converge toward the leaf apex [28]. In order to examine characteristics of the dicotyledonous plant leaves and monocotyledonous plant leaves, the morphology and density of leaf veins and the branch angle between leaf veins were considered in selecting plant species. Three species of dicotyledons with netted venation (including *Populus alba* Linn. (Salicaceae), *Syringa oblata* Lindl. (Oleaceae), and *Ailanthus altissima* (Mill.) Swingle (Simaroubaceae)) and three species of monocots with parallel venation (including *Canna indica* Linn. (Cannaceae), *Hosta plantaginea* (Lam.) Aschers. (Liliaceae), and *Rhapis excelsa* (Thunb.) Henry ex Rehd. (Palmae)) were collected at random from Jilin University in Changchun, China. As shown in Figure 1, *P. alba* has palmate netted venation and both *S. oblata* and *A. altissima* have pinnate netted venations. *C. indica*, *H. plantaginea*, and *R. excelsa* have penni-parallel, campylodromous, and parallelodromous venation, respectively. All sampled leaves were fully developed without any visual damage or senescence. The samples were wrapped in inert waterproof plastic film and stored at 4°C to avoid loss of turgor pressure before the observation and mechanical measurements. Observations and measurements were performed within a day of the collection.

### 2.2. Measurement of Leaf Traits

Leaf traits have a major impact on leaf macroscale mechanical behavior. In order to measure leaf size, leaf shape, and major vein traits of leaves, whole images of leaves of six plant species were captured using a digital camera (Model Nikon D7500, Tokyo, Japan) and manually analyzed by the image processing software *Digimizer*. Whole leaf images were measured for leaf area; maximum leaf length/width as an index of shape; lengths of 1°, 2°, and 3° veins (or midrib and lateral veins) [9]; and branching angle. The vein density (mm mm^−2^) was expressed as the sum of the length of all its segments (mm) per unit area (mm^2^) [2]. Vein lengths of netted venation plants *A. altissima*, *S. oblata*, and *P. alba* were measured for all 1° veins and for 2° veins on one-half of the leaf and doubled for the total 2° vein length. The density of 3° veins was averaged for one to three subsampled regions measured centrally in the top, middle, and bottom thirds of the right side of the leaf. For parallel venation plants, vein lengths of *H. plantaginea* and *R. excelsa* were measured for all main veins from the base. Vein lengths of *C. indica* were measured from midrib veins, and the density of lateral veins was averaged for one to three subsampled regions measured centrally in the top, middle, and bottom thirds of the right side of the leaf. Angles made by the secondary veins to the midvein were measured on the distal side of the junction (the vertex) between the secondary vein and the midvein. Following Ellis et al. [28], each angle was determined as the angle subtended by two elements or rays. One ray followed the midvein distal to the junction and the other intersected the secondary 25% along the length of the secondary. The branching angle between 2° and 3° veins was also measured in the same way.

### 2.3. Tensile Tests and Deformation Behavior of Leaves

Tensile tests were performed on the leaf samples using a self-developed mechanical testing apparatus (Model Mtest50, China). The experiments were conducted at a strain rate of 3.0 mm/min at room temperature. In the preparation of tensile samples, we were primarily concerned with the angle between the direction of the relevant major vein and the direction of the tensile force on the sample. Unless indicated, the width of all samples was 4.5 mm and the length was 30 mm. All samples were excised by a pair of parallel razor blades. According to the distribution and orientation of veins, four samples of netted venation leaves and three samples of parallel venation leaves were taken for tensile tests, as shown in Figure 1. For netted venation leaves, sample 1 includes the midrib which was cut distally from a point 2 cm from the base of the leaf with the long axis parallel to the midrib. Sample 2 was cut parallel to and 2 cm lateral to the midrib. The sample was taken halfway along the leaf. In sample 3, the long axis of the sample was perpendicular to the 2° vein so that the tensile force was applied at right angles to the vein. The sample was taken halfway along the 2° vein that diverged from the midrib in the centre of the leaf. Sample 4 is the intercostal lamina (lamina between secondary veins). Sample 4 was cut parallel to the 2° vein and taken halfway along the 2° vein that diverged from the midrib in the centre of the leaf. For parallel venation leaves, all samples are taken in the middle of the whole leaf. The diameter of midrib of *C. indica* is large and cannot be measured under the same test conditions, so the midrib is not considered. In order to get the same direction of the major vein in the tensile samples of three parallel vein plants, the midrib of the *H. plantaginea* leaf is not considered. The 1° vein or lateral vein is parallel to the tensile direction in sample 1. The samples 2 of *H. plantaginea* and *C. indica* were cut parallel to and 2 cm lateral to the midrib. There is an acute angle between the long axis of the sample 2 and the major vein, so the tensile force was applied at the vein with acute angles. In order to get the same direction of the major vein in the sample 2 of three parallel vein plants, an angle of approximately 45° was set between the major vein and the tensile direction in sample 2 of *R. excelsa*. The 1° vein or lateral vein is perpendicular to the tensile direction in sample 3. The leaf of *R. excelsa* is very narrow, and its tensile samples contain all levels of veins.

Since *A. altissima* leaves have a thicker 1° vein, the width of sample 1 was cut to 10 mm to observe the propagation of cracks better. It is noted that only one tensile sample was excised from each leaf of *S. oblata* and *P. alba*. Each sample was excised just before the measurement so that the test sample was kept as fresh as possible. The mean values were calculated and recorded for three samples for each species.

Vincent [29] suggested that the tensile sample can avoid necking if the test piece was eight to ten times longer than its width and that the tensile test on a notched specimen can provide both the work to fracture (toughness) and information about the ease of propagating a crack through the material. However, the samples were prepared differently from above in this paper. We were interested in the effect of vein distribution on mechanical properties, deformation behavior, and crack propagation behavior of leaves of three dicot and three monocot plant species. When leaves with different vein distributions were subjected to tensile forces, the initiation position and propagation path of cracks are important. Because of the irregular nature of the test samples, it is not appropriate to use the long size of an effective sample (initial distance between clamps). The results of mechanical properties obtained from the leaf will be different because the whole edge and the supporting part of the leaf are destroyed. Meanwhile, if the material is “notch-sensitive,” the crack will potentially have a dangerous effect on the integrity of the material since a small crack may severely lead to the whole material being broken. Therefore, we did not use a notch in the leaf sample as we did not want to control where the first crack appears. Our measurements may have some variation, but we maintain that our test conditions are better for comparing mechanical properties among the different test pieces.

In the tensile tests, the samples were clamped by a pair of clamps, and the free length between the clamps was about 12 mm. A pair of rubber pads was fixed on the clamps of the testing machine in order to prevent the samples from being damaged by the clamps. The thickness and width for all leaves were measured using a Pro-Max digital caliper (Fowler Instruments, Boston, MA, USA), and then the cross-sectional areas of the samples were calculated. The force-displacement curves were recorded automatically. The linear portion of the curve, prior to the catastrophic failure of the tissue, was used to calculate the maximum slope. The tensile strength (*σ*), elastic modulus (*E*), and elongation at fracture (*ε*) are calculated by

(1) $$\begin{array}{c} \text{Tensile strength }\left(\sigma \right)=\frac{F}{A},\\ \text{Elastic modulus }\left(E\right)=\frac{\Delta F/A}{\Delta l/l_{0}},\\ \text{Elongation at fracture }\left(\varepsilon \right)=\frac{\Delta l}{l_{0}}\times 100\%, \end{array}$$

where *F* is the maximum load (N), *A* is the cross-section area of the sample (mm^2^), *l*_0_ is the original length of the sample between the clamps, and Δ*l* is the displacement (mm).

A digital microscope (B011, Supereyes, China) was employed to capture the deformation behavior of the leaf during the tensile test, which was also used to obtain the low-power microimage of leaf morphology after the test. According to the images of these leaf morphologies, the effect of vein distribution on the deformation behavior and fracture mechanisms of leaves of six plant species was analyzed.

## 3. Results and Discussion

### 3.1. Leaf Traits of Leaves of Six Plant Species

**Figure 2:**
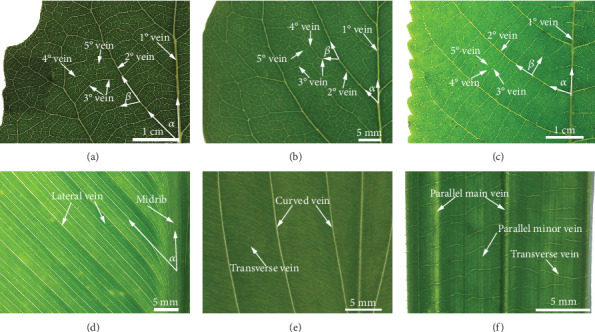
Vein traits of leaves of three dicot and three monocot plant species: (a) *P. alba*, (b) *S. oblata*, (c) *A. altissima*, (d) *C. indica*, (e) *H. plantaginea*, and (f) *R. excelsa*.

Vein traits of leaves of three dicot and three monocot plant species are shown in Figure 2, and the structural parameters of the leaf morphology and vein traits are listed in Table 1. Compared with the three species of dicotyledons with netted venation, three species of monocots with parallel venation have higher leaf length-to-width ratio values. Moreover, except for the leaves of *C. indica*, the thickness of the leaves of the other five species does not differ significantly.

The leaves with netted venation have the greatest diversity in vein structure but share key architectural elements, that is, a hierarchy of vein orders forming a reticulate mesh. As shown in Figures 2(a)–2(c), at least five levels of veins with different diameters appear in the venation system of three species of netted venation plants. Midvein spreads from the petiole to the apex and grows with the lamina. The secondary veins start from midvein and run in parallel towards the margin, and higher veins reconnect with the lower ones. In the hierarchical leaf vein system of angiosperms, veins of higher branching orders in a given leaf have smaller diameters but greater branching frequencies and lengths. The densities (length/leaf area) of the major veins show declines in larger leaves. *P. alba* with palmate netted venation possesses the highest 1° vein densities. The leaf size of *A. altissima* was larger than that of *S. oblata*. However, the 1° and 2° vein densities of *A. altissima* were smaller than those of *S. oblata*. Noteworthy, the density change of the 3° vein was not obvious between three species of netted venation plants. Quaternary and quinary veins of netted venation plants cover most of the leaf area whilst having the smaller diameter.

Vein branching angles are also diverse across species. In mature leaves, venation patterns are extremely diverse, yet their local structure satisfies a universal property: at junctions between veins, angles and diameters are related by a vectorial equation analogous to a force balance [12]. This vectorial equation can be expressed in the whole vein system; the angle within each three-way vein junction is proportional to the radii of the connecting veins [1, 30]. For that reason, the vector balance criterion gives proper angles existing in vein branches in the leaves of same plant species. The branching angles between secondaries and the major vein of *P. alba*, *S. oblata*, and *A. altissima* varies from 41° to 76°, 43° to 65°, and 53° to 67°, respectively. And their mean angles were 61°, 53°, and 66°, respectively. Similarly, the mean branching angles between secondaries and the major vein of *P. alba*, *S. oblata*, and *A. altissima* were 76°, 74°, and 86°, respectively. It can be found that the vein branches are usually connected by the shortest path. In the larger plant leaves, tertiary veins are parallel to each other and approximately perpendicular to the secondary veins.

In leaves with parallel venation, the veins are either strictly parallel, curved and approximately parallel, or penni-parallel, as shown in Figures 2(d)–2(f). *C. indica* has dense and thin parallel secondary veins branching off the midrib. *H. plantaginea* possesses campylodromous veins, and thin transverse veins connect adjacent campylodromous veins. *R. excelsa* possesses parallel veins and thin transverse veins. Due to the presence of transverse veins, these leaves form a gridded system that is similar to that of dicotyledons. Large and intermediate longitudinal veins are analogous to major vein orders, and small longitudinal veins and transverse veins are analogous to minor veins [31]. The small transverse veins in these leaves reinforce against bending forces [1]. Moreover, inherent folding or curling in the *R. excelsa* leaf can also contribute to the structural stiffness [22]. The density of midribs or major veins of parallel venation leaves also decreases with increasing leaf area.

Although the leaves of the six species of plants have different venation systems, the veins show common hierarchy characteristics. Hierarchy and network characteristics of leaves guarantee the global rigidity and local isotropy that are required in the reinforcement layout [32]. In order to further study the effect of vein distribution on the mechanical behavior, the tensile properties of different leaf venation systems were studied by in situ tensile tests in the following sections.

### 3.2. Mechanical Properties

**Figure 3:**
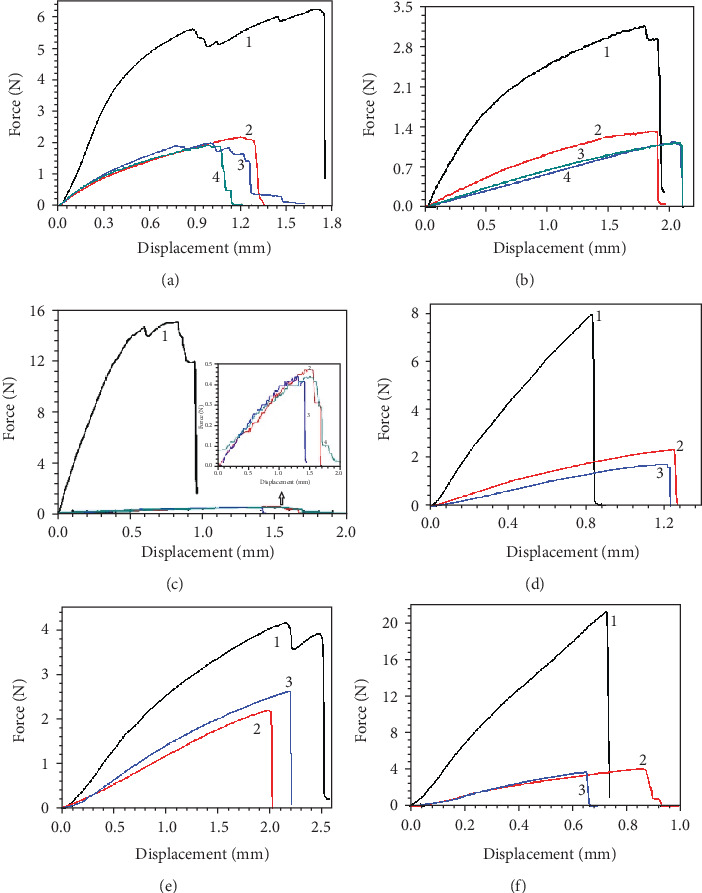
Typical load-displacement curves of tensile tests on leaves of three dicot and three monocot plant species: (a) *P. alba*, (b) *S. oblata*, (c) *A. altissima*, (d) *C. indica*, (e) *H. plantaginea*, and (f) *R. excelsa*.

The typical force-displacement curves of tensile tests on leaves of three dicot and three monocot plant species are plotted in Figure 3, where the numbers 1-4 are the locations on the leaf of the four test samples. Beyond the maximum force, several step-like force drops are recorded in netted venation plants and the sample 1 of *H. plantaginea* with parallel veins, which correspond to successive fracture behaviors of veins. As shown in Figures 3(a)–3(c), when leaves of *P. alba*, *S. oblata*, and *A. altissima* with netted venation were stretched from tensile forces, the force-displacement curve displays the elastic-plastic type [25]. The descending curve is ragged, which indicates that the veins break sequentially in the leaves as the load increases. It is noted that *H. plantaginea* possesses soft leaves and low vein density, and the tensile curves are similar to netted venation leaves. On the contrary, the force-displacement curves of *C. indica* and *R. excelsa* show no clear plastic phase before fracture and behave in a predominantly brittle manner [23]. Additionally, the descending curve following catastrophic failure of the leaves remained linear, indicating that the majority of the veins responsible for the tensile strength were breaking at about the same time. The toughness is defined as the work to fracture, measured as the area under the force-displacement curve [19]. In Figures 3(a)–3(c), the toughness of sample 1 is higher than that of sample 2, and sample 4 is the lowest in the three species of netted venation leaves. As shown in Figures 3(d)–3(f), the similar patterns are also shown in parallel venation leaves. But, the sample 3 of *H. plantaginea* is tougher than sample 2.

**Figure 4:**
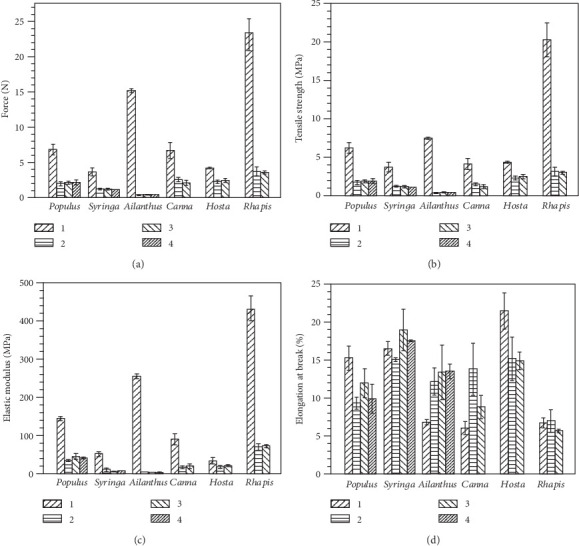
Tensile properties of leaves of three dicot and three monocot plant species: (a) maximum load, (b) tensile strength, (c) elastic modulus, and (d) elongation at fracture.

The variations in maximum load, tensile strength, elastic modulus, and elongation at the complete fracture of leaves of three dicot and three monocot plant species in different test location of leaves are plotted in Figure 4, where the numbers 1-4 indicate the location on the leaves of the test samples. There are significant differences among species for each of the tensile tests. However, the values of maximum load, tensile strength, and elastic modulus of sample 1 of all six species are the largest. In particular, *A. altissima* possesses a larger main vein diameter and thinner leaf thickness than *P. alba* and *S. oblata*. The tensile strength and elastic modulus of the main vein sample 1 of *A. altissima* were 16 and 52 times larger than those of samples 2-4, respectively. However, the maximum load, tensile strength, and elastic modulus of samples 2-4 of *A. altissima* were the smallest among those of the six species of plant leaves. Similarly, the maximum load, tensile strength, and elastic modulus of sample 1 of *R. excels* were 6 times larger than those of samples 2 and 3. Except for sample 1, samples 2 and 3 of the three species of monocots with parallel venation have higher maximum load, tensile strength, and elastic modulus values when compared to samples 2-4 of the three species of dicots with netted venation. It is noted that the differences of maximum load, tensile strength, and elastic modulus of samples 2-4 of the same species were not significant.

The distribution and orientation of veins have a significant influence on the tensile properties of leaves. The venation has better mechanical performance and antistretch ability compared with other tissues [20]. The main veins and parallel lateral veins bear the main load in plant leaves, which can provide a frame for the lamina to curl, or to allow flexural bending, so as to reduce transpiration and mechanical load [1]. The thin veins of leaves make a relatively small contribution to the mechanical properties of leaves. The greater the diameter and the more the number of veins parallel to the tensile direction, the larger the tensile force, tensile strength, and elastic modulus of the leaves.

The elongation at complete fracture among the six species of plant leaves is significantly different. In addition, elongation before fracture of *C. indica* with dense parallel veins is smaller than that of *P. alba*, while elongation at complete fracture of *H. plantaginea* is larger than that of *P. alba*. The results show that the tensile properties of leaves are related not only to vein direction but also with the material composition and structure of leaves. *R. excelsa* leaves are stronger, tougher, and stiffer than other soft leaves, and elongation at complete fracture of leaves is smaller. In softer leaves, the major veins can improve the tensile strength and elastic modulus, while ensuring that the leaves have a higher elongation at complete fracture. Based on the above research, the mechanical deformation behaviors of leaves need to be discussed.

### 3.3. Mechanical Deformation Behavior

#### 3.3.1. Crack Propagation Behavior of Leaves with Netted Venation

**Figure 5:**
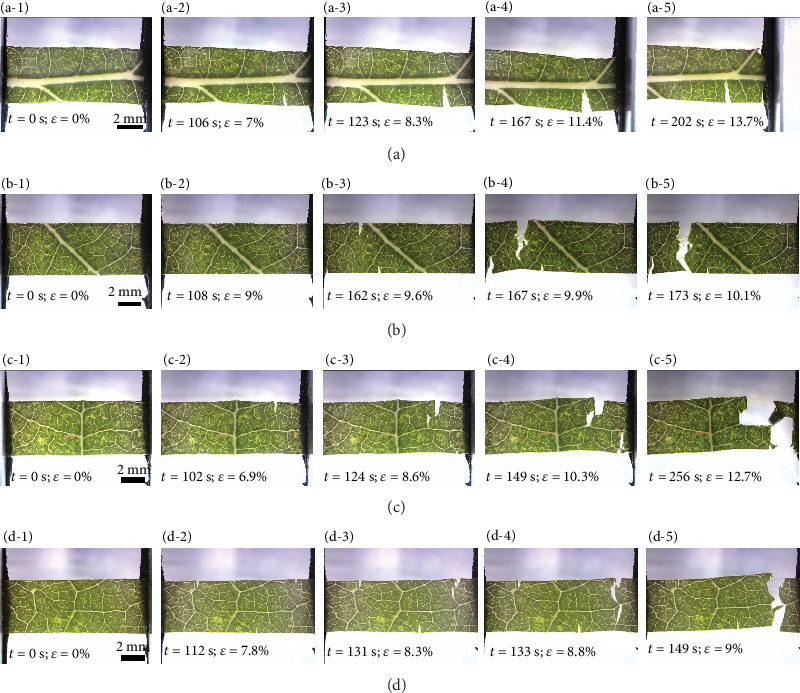
Fracture growth in ((a-1)–(a-5)) sample 1, ((b-1)–(b-5)) sample 2, ((c-1)–(c-5)) sample 3, and ((d-1)–(d-5)) sample 4 with different vein distributions of *P. alba* leaves.

The crack propagation behavior of leaf samples with different vein patterns and distribution of *P. alba* in tensile tests is shown in Figure 5. The time marked in Figure 5 represents the time of the whole crack propagation process. The corresponding first figure in (a-1)–(a-5), (b-1)–(b-5), (c-1)–(c-5), and (d-1)–(d-5) is the initial state. The second figure shows the initiation of cracks. The third and fourth figures show the propagation of cracks. The fifth figure reflects the final fracture state. The crack propagation of vein direction parallel to the tensile direction of sample 1 is shown in Figures 5(a-1)–5(a-5). With elongation going on, sample 1 gradually reached the maximum elastic elongation of 7% at the beginning of 106 s, and cracks occur at a zone of weakness at the mesophyll in Figure 5(a-2). Then, the crack continues to propagate in the mesophyll until the crack tip encounters the main vein and the crack stops propagating in Figures 5(a-3) and 5(a-4). Finally, the main veins and mesophyll snap at the gripping end long after the mesophyll separated. Samples 2 and 3 possess 2° and 3° veins; as elongation increases, cracks initiate in the mesophyll. The cracks deviate frequently, when the crack tip encounters the 2° and 3° veins in Figures 5(b-3)–5(b-5) and 5(c-3)–5(c-5). The tensile curve in Figure 3(a) also supports this observation. The mesophyll or small veins were breaking first, and due to the frequent deflection of the cracks, the descending curve was ragged. Thin veins play a minor role in preventing the propagation of cracks. As shown in Figures 5(d-3)–5(d-5), the cracks in the intercostal leaf sample 4 propagate through the thin veins until the leaf finally breaks.

**Figure 6:**
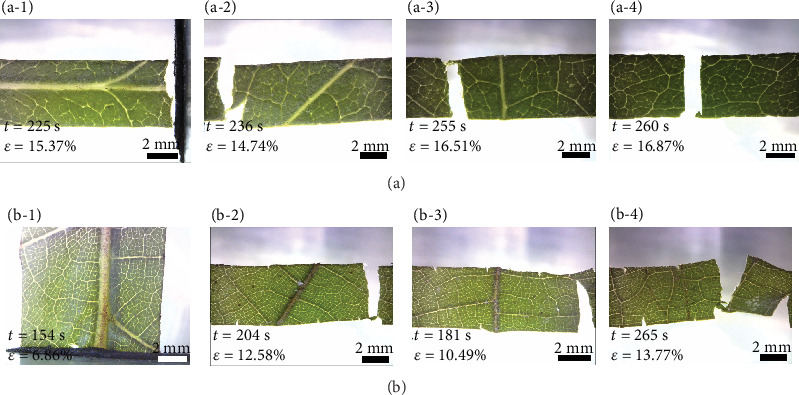
Microscopic images of the fracture on the leaves with different vein distributions: ((a-1)–(a-4)) *S. oblata* and ((b-1)–(b-4)) *A. altissima*.

In Figure 6, *S. oblata* and *A. altissima* exhibit similar crack behaviors. The leaves of *S. oblata* are softer than *P. alba*, and the veins of *S. oblata* are weak. In addition to sample 1 with the main veins, the crack does not deflect obviously in samples 2-4 of the leaves without the main veins. Because *A. altissima* has a larger main vein diameter and thinner leaf thickness, the main vein of sample 1 bears the main load, and the sample breaks directly at the clamping end. The crack propagation behaviors of samples 2-4 are similar to *S. oblata*. The results show that the 3° veins and thin veins of *S. oblata* and *A. altissima* play a minor role in the propagation of crack process.

The hierarchy and network venation in netted venation leaves have a significant effect in the crack propagation process. The leaves with netted venation exhibit ragged breakage patterns indicating that tissues of the leaf have different abilities to resist tensile stress. Cracks are usually produced in the mesophyll and deflected, delayed, or even stopped once reaching the main vein or 2° vein. Some of the stronger 3° veins can also cause crack deflections, but most of the thin veins are weak and have no noticeable effect on crack propagation.

#### 3.3.2. Crack Propagation Behavior of Leaves with Parallel Venation

**Figure 7:**
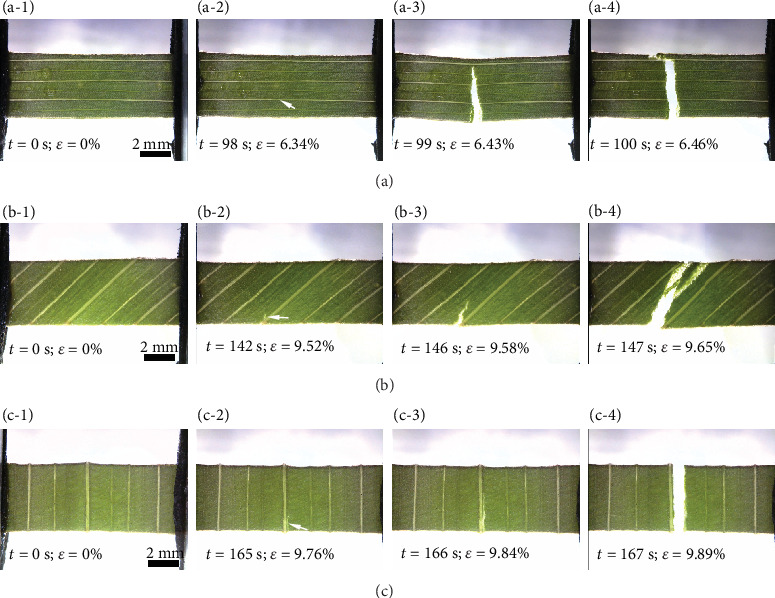
Fracture growth in ((a-1)–(a-4)) sample 1, ((b-1)–(b-4)) sample 2, and ((c-1)–(c-4)) sample 3 with different vein distributions of *C. indica* leaves. The white arrows in (a-2), (b-2), and (c-2) show the crack initiation sites.

The crack propagation behaviors of leaf samples with different vein distributions in *C. indica* are shown in Figure 7. The corresponding first figure in (a-1)–(a-4), (b-1)–(b-4), and (c-1)–(c-4) is the initial state. The second figure shows the initiation of cracks, and white arrows show the crack initiation site. The third figure illustrates crack propagation. The fourth figure reflects the final fracture state. The crack propagation in samples where the vein direction is parallel to the tensile direction is shown in Figures 7(a-1)–7(a-4). With elongation, the sample 1 specimen gradually reached its maximum elastic elongation, 6.34% over the initial length at 98 s, in a main vein as shown in Figure 7(a-2). The cracks propagate rapidly through the adjacent mesophyll in two directions (Figure 7(a-3)) with almost instantaneous failure of the adjacent main veins with only a further 0.12% increase in elongation over 2 s for the complete fracture (Figure 7(a-4)).

During the stretching, the cracks propagate through the parallel veins in sample 1, so a larger tensile load needs to be loaded on the leaf to promote crack initiation. Compared with sample 1, the cracks in samples 2 and 3 are produced at the interface of the veins and the mesophyll and then propagate and break almost instantaneously along the mesophyll or interface, so the load at the break of samples 2 and 3 is smaller than that of sample 1. Although the breaks almost appear instantaneously, we also observed that the fracture starts at the interface of one vein but ends at the interface of another small longitudinal vein in sample 2. Additionally, most veins responsible for the tensile strength were breaking at about the same time. Fracture may also be dependent on the velocity of elongation not allowing the propagating crack to be deflected at the interface of the next vein to fracture. This phenomenon also verifies that the descending curve following catastrophic failure of the leaves remained linear in Figure 3(d). Although the maximum loads of samples 2 and 3 are reduced by the changes of distribution direction of the veins, they increase the elongation at the complete fracture of the sample.

**Figure 8:**
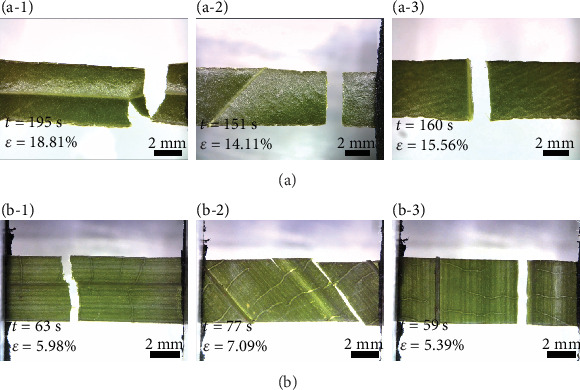
Microscopic images of the fracture on the leaf with different vein distributions: ((a-1)–(a-3)) *H. plantaginea* and ((b-1)–(b-3)) *R. excelsa*.

*H. plantaginea* possesses soft leaves and low vein density (Figures 8(a-1)–8(a-3)), and its crack propagation behaviors are similar to netted venation leaves. There are a lot of thin transverse veins between campylodromous veins in the leaves of *H. plantaginea*, but when tensile load is put on the leaf, the transverse veins provide little resistance. Therefore, the crack is usually produced in the mesophyll or at the interface between the mesophyll and the veins and then rapidly propagates until the leaf fractures. The crack propagation behavior of highly scleromorphic *R. excelsa* leaves is similar to that of *C. indica* leaves, as shown in Figures 8(b-1)–8(b-3). However, the leaf has a greater density of veins, and the change of the distribution and orientation of veins has little effect on the elongation at the complete fracture of the sample. Most of the transverse veins are weak and have no noticeable effect on crack propagation.

Parallel venation has a significant effect on the process of crack propagation. Parallel veins increased the force required to fracture leaves in tensile tests by spreading the load across many strong veins thereby inhibiting the initiation of cracks. However, once a crack has initiated in one vein and it breaks, the current load is suddenly spread across the remaining veins, exceeding their individual strength, causing the whole leaf to fail catastrophically. In the case of leaves with parallel veins, the mesophyll, weak as it is, may act sufficiently to prevent remaining veins from sliding and individually accommodating the increased load consequent on failure of one vein, leading to catastrophic failure. Therefore, a material can be designed to have parallel veins with slightly different strengths, rather than the same strength, which may make the material tougher. By adjusting the spacing between parallel veins, the fracture of the materials can be controlled more easily.

The results of vein traits, mechanical tests, and deformation behavior show that the tensile properties, deformation behavior, and crack propagation behavior of plant leaves are related to the stiffness of the leaves, the degree of development of the veins, and the distribution and orientation of the veins in the leaves during tensile tests. The leaves with netted venation evince the elastic-plastic fracture type, and a hierarchy of different venation networks suppresses the propagation of cracks in the leaves by deflecting, delaying, or even stopping the crack in the leaves. Most of the leaves with parallel venation behave in a predominantly brittle manner, and parallel veins increased the force required to fracture leaves in tensile tests by spreading the load across many strong veins thereby inhibiting the initiation of cracks.

## 4. Conclusions

In this study, the vein traits and mechanical properties of six species of plant leaves with different leaf venation systems were investigated. Based on the leaf morphology and vein traits of the leaf, several typical test locations from each leaf were selected. The tensile properties and the deformation behavior were studied using quasi in situ tensile testing apparatus under a digital microscope. According to the vein traits, mechanical tests, and deformation behavior results, it was found that the vein distribution of leaves had a remarkable effect on their mechanical properties, deformation behavior, and crack growth. The results can be summarized as follows:

1. Leaves have an excellent hierarchy of veins and network characteristics, which supports the stiffness of the whole leaf. The density of major veins decreases with increasing leaf area, and the vein branches usually take the shortest path to connect. A branching angle of 41°~75° was observed between the 1° and 2° veins, and near 90° between higher veins in dicotyledons. Leaf veins of monocotyledons are usually parallel to each other, or there are small transverse veins perpendicular to them
2. In situ tensile experiments show that the vein architecture has a remarkable effect on their mechanical properties. The greater the diameter and the more the number of veins parallel to the tensile direction, the higher the tensile force that can be resisted and the overall higher tensile strength and elastic modulus of the leaves. Compared with netted venation species, leaves of parallel venation species show comparatively high tensile stiffness. The leaves with netted venation evince the elastic-plastic fracture type, and most of the leaves with parallel venation behave in a predominantly brittle manner
3. The results of crack propagation verify the differences in the force-displacement curves in the situ tension test leaves of six plant species. The effectiveness of resisting crack propagation is related to the stiffness of the leaves, the degree of development of the veins, and the distribution and orientation of the veins in the leaves during tensile tests. The hierarchy network venation lengthens the crack path and provides resistance to the fracture propagation. The parallel venation increased the load at complete fracture of the leaves and inhibits the initiation of cracks
4. Inspired by the branch pattern of the leaf, a material can be designed to have multilevel reticulated veins and/or parallel veins with slightly different strengths, which may make the material tougher

## Figures and Tables

**Table 1:** Leaf morphology and vein traits of six typical plant leaves.

	Netted venation plant			Parallel venation plant		
	*P. alba*	*S. oblata*	*A. altissima*	*C. indica*	*H. plantaginea*	*R. excelsa*
Family	Salicaceae	Oleaceae	Simaroubaceae	Cannaceae	Liliaceae	Palmae
Leaf length (cm)	9.21	10.28	23.48	46.39	13.90	28.10
Leaf width (cm)	8.42	7.54	9.55	17.44	7.69	1.69
Leaf length-to-width ratio	1.09	1.36	2.46	2.66	1.81	16.63
Leaf thickness (mm)	0.24	0.21	0.19	0.35	0.22	0.25
Leaf area (cm^2^)	51.21	49.57	168.95	531.38	75.58	34.99
1° vein density (mm mm^−2^)	0.03	0.02	0.01	0.01	0.22	0.38
2° vein density (mm mm^−2^)	0.08	0.11	0.09	0.6	—	—
3° vein density (mm mm^−2^)	0.3	0.27	0.29	—	—	—
Angle between 1° and 2° veins (*α*)	41°-76°	43°-65°	53°-75°	21°-59°	—	—
Angle between 2° and 3° veins (*β*)	63°-86°	62°-90°	65°-90°	—	—	—

## Data Availability

The data used to support the findings of this study are included within the article.
